# Template copy number and the sensitivity of quantitative PCR for *Plasmodium falciparum* in asymptomatic individuals

**DOI:** 10.1186/s12936-020-03365-8

**Published:** 2020-08-18

**Authors:** Trevor A. Thompson, Mahamoudou B. Touré, Daouda Sanogo, Jeffrey G. Shaffer, Seydou O. Doumbia, Donald J. Krogstad

**Affiliations:** 1 West African International Center of Excellence for Malaria Research, Bamako, Mali; 2 Tulane School of Public Health and Tropical Medicine, 1430 Tulane Avenue, #8317, J.B. Johnston Building, Room 510, New Orleans, LA 70112-2699 USA; 3 University of the Sciences, Techniques and Technologies of Bamako, Bamako, Mali

**Keywords:** *Plasmodium falciparum*, Asymptomatic, Template copy number, Quantitative PCR (qPCR), Diagnostic

## Abstract

**Background:**

The identification of asymptomatic individuals with *Plasmodium falciparum* infection is difficult because they do not seek medical treatment and often have too few asexual parasites detectable using microscopy or rapid diagnostic tests (≤ 200 parasites per μl). Quantitative PCR (qPCR) may provide greater sensitivity and permits estimation of the initial template DNA concentration. This study examined the hypothesis that qPCR assays using templates with higher copy numbers may be more sensitive for *P. falciparum* than assays based on templates with lower copy numbers.

**Methods:**

To test this hypothesis, ten qPCR assays for DNA sequences with template copy numbers from 1 to 160 were compared using parasite DNA standards (*n *= 2) and smear-positive filter paper blots from asymptomatic smear-positive subjects (*n *= 96).

**Results:**

Based on the testing of *P. falciparum* parasite DNA standards and filter paper blots, cycle threshold values decreased as the concentrations of template DNA and template copy numbers increased (*p *< 0.001). Likewise, the analytical and clinical sensitivities of qPCR assays for *P. falciparum* DNA (based on DNA standards and filter paper blots, respectively) increased with template copy number. Despite the gains in clinical sensitivity from increased template copy numbers, qPCR assays failed to detect more than half of the filter paper blots with low parasite densities (≤ 200 asexual parasites per μl).

**Conclusions:**

These results confirm the hypothesis that the sensitivity of qPCR for *P. falciparum* in the blood of individuals with asymptomatic infection increases with template copy number. However, because even the most sensitive qPCR assays (with template copy numbers from 32 to 160) detected fewer than 50% of infections with ≤ 200 asexual parasites per μl, the sensitivity of qPCR must be increased further to identify all smear-positive, asymptomatic individuals in order to interrupt transmission.

## Background

As malaria control decreases plasmodial infection and malaria disease, the inadequate sensitivity of current methods for *Plasmodium falciparum* becomes an increasingly important limitation [1]. Although asymptomatic individuals harbour a substantial fraction of the parasites in the community [2–4], those individuals do not seek care because they are not sick and often have too few asexual parasites detectable using microscopy or rapid diagnostic tests (RDTs, ≤ 200 per μl). However, because asymptomatic parasitized individuals can infect mosquitoes [5–7], the failure of current methods to identify asymptomatic infected individuals is an obstacle to the interruption of transmission and thus to the improvement of malaria control strategies necessary for its ultimate elimination.

In contrast to conventional diagnostic methods, PCR-based methods permit the exponential amplification of parasite DNA or RNA to detect *P. falciparum*. For this reason, PCR-based methods have the potential to provide greater sensitivity than microscopy or RDTs [8]. However, the information available about the sensitivity of PCR-based methods for *P. falciparum* in asymptomatic individuals and the factors affecting their sensitivity is limited. Although the sensitivity of PCR-based methods for *P. falciparum* is thought to depend on the template copy number [9, 10], the effects of the template copy number on the sensitivity of PCR-based methods for *P. falciparum* in asymptomatic individuals have not been examined systematically—by using similar PCR-based methods grouped by their template copy numbers.

PCR-based methods used to detect *P. falciparum* include but are not limited to: (1) conventional PCR, (2) nested PCR and (3) quantitative PCR (qPCR). Of those strategies, only qPCR permits estimation of the initial template DNA concentration from the cycle threshold (C_t_) value—the number of amplification cycles required for the fluorescent signal to exceed the threshold level of background fluorescence. For that reason and because DNA is more stable than RNA, these studies emphasize the use of qPCR amplification of parasite DNA to detect *P. falciparum* in asymptomatic individuals.

To examine the effects of template copy number on the sensitivity of qPCR, we compared qPCR assays with template copy numbers from 1 to 160 for the detection of *P. falciparum* DNA in laboratory standards and smear-positive filter paper blots from asymptomatic infected human subjects. The hypothesis underlying these studies was that the sensitivity of qPCR should increase with the template copy number of the qPCR assay.

## Methods

### In silico development and screening of candidate qPCR assays

The qPCR assays examined initially were based on PubMed literature searches for template copy numbers and qPCR assays for *P. falciparum*. Additional candidate assays were then developed using the AlleleID^®^7 software from Premier Biosoft (Palo Alto, CA). Each candidate assay was screened using NCBI Primer-BLAST [11] to: (1) estimate its template copy number (number of DNA sequences yielding a positive fluorescent signal) based on the *P. falciparum* 3D7 genome (ASM2762), (2) examine the AT content of DNA sequences amplified and (3) test for cross-reactivity with host or pathogen DNA. In contrast, because the template copy numbers for mitochondrial DNA sequences cannot be estimated in silico, representative median estimates of mitochondrial genome copy numbers were used for the *cytb* and *coxI* assays [12].

### Effects of different qPCR reagents and conditions on assay performance

Based on the studies described above, 13 candidate qPCR assays were selected for laboratory testing (Table 1) [13–19]. Primers and probes were synthesized by Integrated DNA Technologies (Coralville, IA) using additional modifications as noted (Table 2). These pilot studies and the subsequent studies were performed using a BioRad iQ5™ real-time instrument (Hercules, CA) with 96 well microtitre trays and analysed using the BioRad iQ5™ Optical System Software (v 2.1).

**Table 1:** Template copy number estimates for 13 candidate qPCR assays

qPCR assay	References	*P. falciparum* DNA sequence	Template copy No.^a^
*crt*	This article	Chloroquine-resistance transporter	1
*crt*76 K	[13]	Chloroquine-resistance transporter (76K)	1
*crt*76T	[13]	Chloroquine-resistance transporter (76T)	1
*ldh*(a)	[14]	Lactate dehydrogenase	1
*ldh*(b)	This article	Lactate dehydrogenase	1
*18SrRNA*(a)	[15]	18S Ribosomal ribonucleic acid	3
*18SrRNA*(b)	[16]	18S Ribosomal ribonucleic acid	3
r364(a)	[17]	Repetitive element 364	14
*cytb*	[18]	Cytochrome b	22
*coxI*	This article	Cytochrome oxidase I	22
*var*ATS	[19]	*var* gene acidic terminal sequence	29
r364(b)	This article	Repetitive element 364	56
TARE-2	[19]	Telomere-associated repetitive element 2	160

^a^ Template copy number estimates for the *P. falciparum* DNA sequences yielding positive fluorescent signals (based on the 3D7 *P. falciparum* genome)

**Table 2:** Design of 13 candidate qPCR assays in ascending order by template copy number

qPCR assay	Fluorescence	Forward primer (FP), reverse Primer (RP) and Probe sequences (5′ → 3′)
*crt*	MGB TaqMan	FP: GACACCGAAGCTTTAATTTAC RP: GCAGAAGAACATATTAATAGGAA Probe: 5′-HEX/TTAGATGCCTGTTC/3′-MGBEc/
*crt*76K *crt*76T	MGB TaqMan	FP: TGGTAAATGTGCTCATGTGTTT RP: AGTTTCGGATGTTACAAAACTATAGT Probe(K76): 5′-HEX/TGTGTAATGAATAAAATTTTTGCTAA/3′-MGBEc/ Probe(T76): 5′-6-FAM/TGTGTAATGAATACAATTTTTGCTAA/3′-MGBEc/
*ldh*(a)	Standard TaqMan	FP: ACGATTTGGCTGGAGCAGAT RP: TCTCTATTCCATTCTTTGTCACTCTTTC Probe: 5′-HEX/AGTAATAGTAACAGCTGGATTTACCAAGGCCCCA/3′-IABkFQ/
*ldh*(b)	MGB TaqMan	FP: TGGTCATATTAAGAAGAATTGTC RP: CTGAGATATGTAATACTTCAATC Probe: 5′-HEX/CCATAACATCTACT/3′-MGBEc/
*18SrRNA*(a)	Standard TaqMan	FP: CTTTTGAGAGGTTTTGTTACTTTGAGTAA RP: TATTCCATGCTGTAGTATTCAAACACAA Probe: 5′-HEX/TGTTCATAACAGACGGGTAGTCATGATTGAGTTCA/3′-IABkFQ/
*18Sr*RNA(b)	MGB TaqMan	FP: ATTGCTTTTGAGAGGTTTTGTTACTTT RP: GCTGTAGTATTCAAACACAATGAACTCAA Probe: 5′-HEX/CATAACAGACGGGTAGTCAT/3′-MGBEc/
r364(a)	PET	FP: ACCCCTCGCCTGGTGTTTTT RP/Probe: 5′-HEX/aggcgcatagcgcctggTCGGGCCCCAAAAATAGGAA
*cytB*	LNA TaqMan	FP: TACTAACTTGTTATCCTCTATTCCAGTAGC RP: CCTTTAACATCAAGACTTAATAGATTTGGA Probe: 5′-HEX/+ GTG + CTA + CCA + TGT + AAA + TGTAA/3′-IABkFQ/
*coxI*	MGB TaqMan	FP: GTCACGCAATATCAATATACTG RP: CGATCTCTTGTATGGTAATAGG Probe: 5′-HEX/ATAGAACTCCAGGC/3′-MGBEc/
*var*ATS	MGB TaqMan	FP: CCCATACACAACCAAYTGGA RP: TTCGCACATATCTCTATGTCTATCT Probe: 5′-HEX/TRTTCCATAAATGGT/3′-MGBEc/
r364(b)	MGB TaqMan	FP: AGTCCATTTTCCCCTAGC RP: GACCATATAGTAAGTGACCCA Probe: 5′-HEX/AATTGACATGCACT/3′-MGBEc/
TARE-2	dsDNA-dye	FP: CTATGTTGCACTTACATGCAYAAT RP: TGACCTAAGAAGTAVAATAATGATGA

Information provided in this table includes the abbreviations for each of the 13 qPCR assays, the source of the fluorescent signal in each assay and the sequences of the forward and reverse primers and probes for each assay (beginning on the left with the 5′ end and concluding on the right with the 3′ end for each primer and probe)

MGB, minor groove binder; PET, photo-induced electron transfer; LNA, locked nucleic acid; dsDNA, double-stranded DNA; 6-FAM, 6-carboxyfluorescein fluorescent dye; HEX, hexachloro-fluorescein fluorescent dye; IABkFQ, Iowa Black fluorescent quencher; MGBEc, MGB Eclipse^®^ fluorescent quencher

To limit the potential for confounding due to using qPCR reagents from only one source, nine assays (*crt*, *crt*76K, *ldh*(a), *18SrRNA*(a), r364(a), *coxI*, *cytb*, *var*ATS and r364(b)) were performed using qPCR reagents available from three different sources: (1) Invitrogen Platinum™ *Taq* with Green PCR buffer (Carlsbad, CA), (2) New England Biolabs (NEB) Luna^®^ Universal Probe qPCR Master Mix (Ipswich, MA) and (3) Quantabio PerfeCTa qPCR ToughMix™ (Beverly, MA). In addition, four factors were examined for their effects on the results obtained with NEB reagents: (1) annealing temperature (T_a_), (2) annealing time, (3) magnesium concentration and (4) final primer and probe concentrations.

### Evaluation of qPCR assays based on parasite DNA standards

Based on the studies above, ten of the 13 candidate assays were examined after removing the *crt*76K, *crt*76T and r364(a) assays. The *crt*76K and *crt*76T assays were removed because they were designed to hybridize with polymorphic DNA sequences for amino acids 72–76 of the *Plasmodium falciparum* chloroquine resistance transporter gene (*pfcrt*) [13]. In contrast, the r364(a) assay was removed because of false-positive results. The other 10 qPCR assays were then compared for their sensitivity and specificity using the controls listed below:

1. Positive *P. falciparum* DNA Controls: eight tenfold dilutions from 10^+3^ to 10^−4^ picograms (pg) DNA per qPCR for 3D7 (drug-susceptible) and Dd2 (drug-resistant) parasite DNA standards provided by the Biodefense and Emerging Infections Research Resources Repository (Manassas, VA),
2. Negative Human DNA Controls: human DNA extracted from filter paper blots of *Plasmodium*-negative, human donor blood collected in the United States and.
3. Negative DNA-Free Controls: nuclease-free water to exclude the contamination of assay reagents or mixtures with parasite DNA.

The qPCR conditions used for the NEB reagents in these studies were considered optimal based on the results of pilot studies examining their effects on assay performance. Thermocycler protocols began with 5 min of denaturation at 95° C followed by 45 cycles of two steps: [1] 30 s for denaturation at 95^o^ C and [2] 60 s for annealing and extension at 50^o^ C (*crt*, *crt*76T, *ldh*(b), *coxI*, *var*ATS and r364(b) assays) or 60° C (*crt*76K, *ldh*(a), *18SrRNA*(a), *18SrRNA*(b), r364(a), *cytb* and TARE-2 assays). This testing was performed using final reaction volumes of 15 µl with forward and reverse primers (400 nm final primer concentrations) and probes (200 nM final probe concentrations). A double-stranded DNA intercalating dye (Lumiprobe dsGreen—Hunt Valley, MD) was added to the TARE-2 assay as suggested by the manufacturer and melt curve analyses were performed as described previously [19]. Thresholds of detection were set for each assay at the point where amplification became exponential (the curve became linear) for the positive controls and C_t_ values ≤ 41 cycles were considered positive.

### Longitudinal population-based study design, sample collection and smear surveys to identify asymptomatic persons with *P. falciparum* infection

Children and adults in Dangassa, Mali were enrolled in the population-based, longitudinal cohort studies of the West African International Centers of Excellence for Malarial Research (ICEMRs) at the beginning and end of the rainy season with their informed assent and their parents’ written informed consent after the study protocol had been reviewed and approved by the NIH (NIAID), the Mali and Tulane Institutional Review Boards (FWA000001769, FWA000002055) and the chief and elders of the village. Enrolled subjects then provided 2 drops of blood (~ 50 μl per drop) with a sterile disposable lancet (as recommended by the Mali Ministry of Health) after the tip of the finger had been cleaned with alcohol and dried. Please note because the results of those studies have been described previously [20], the methods described in this section are provided as clinical context for the human blood samples examined in these molecular studies.

The first drop of blood was applied to a freshly cleaned glass slide to produce a thick smear and the second to a GE Healthcare Life Sciences Whatman FTA filter paper (Pittsburgh, PA) for qPCR. After drying, the thick smear was rinsed in hypotonic phosphate buffer to lyse intact red blood cells before staining with Giemsa to permit the identification of asexual *P. falciparum* parasites by microscopy using oil immersion magnification. Based on 7500 white blood cells per μl as the average white cell count, the number of asexual *P. falciparum* parasites in oil-immersion fields containing 300 white blood cells was multiplied by 25 to estimate the number of asexual parasites per μl of blood [21]. After filter paper blots had been obtained and dried in a covered container, they were stored at ambient temperature in Mali, transported to the U.S., stored at − 20 °C and processed for DNA extraction and laboratory testing in New Orleans.

Afebrile individuals with thick smears positive for asexual *P. falciparum* parasites who had no symptoms or signs of malaria (i.e., no chills, fever, headache, myalgias or arthralgias) were classified as smear-positive asymptomatic persons with *P. falciparum* infection. In contrast, subjects with negative thick smears (no asexual *P. falciparum* parasites in microscopic fields containing 300 white blood cells) and no symptoms or signs of malaria were classified as healthy and uninfected. Members of the cohort who had symptoms or signs consistent with malaria and positive thick smears were examined and treated for their symptoms and signs on the same day without charge.

### Evaluation of qPCR assays based on smear-positive asymptomatic subjects

Based on the filter paper blots for subjects 5–14 years of age collected during thick smear surveys in 2015, 96 filter paper blots from smear-positive asymptomatic subjects were selected randomly to estimate the clinical sensitivities of the qPCR assays. After those specimens had been sorted into groups based on their asexual parasite densities: (1) ≤ 200, (2) 201–999, (3) 1,000–1999 and (4) 2000–5000 per µl, Thermo Fisher Scientific ChargeSwitch^®^ Forensic DNA Purification Kits (Waltham, MA) were used to extract DNA from 3 mm diameter punches of 50 µl blood spots on the filter papers. Six microliters of the 150 µl DNA eluate from each specimen were tested using qPCR. Thermocycler conditions were the same as those for the parasite DNA standards.

### Testing parameters and statistical analyses

The effects of template copy number were examined after grouping the qPCR assays by their mean template copy numbers: [a] 1 copy (*n *= 3 assays: *crt, ldh*(a) and *ldh*(b)), [b] 3 copies (*n *= 2 assays: *18SrRNA*(a) and *18SrRNA*(b)), [c] 32 copies (*n *= 4 assays: *cytb, coxI,* r364(b) and *var*ATS) and [d] 160 copies (*n *= 1 assay: TARE-2). Based on the parasite DNA standards and filter paper blots which yielded positive results (C_t_ values ≤ 41) for each of the 10 assays examined, 2-way ANOVA testing was performed in R Studio [22] to examine the effects of: [a] template copy number, [b] the initial template DNA concentration and [c] interactions between those factors and the final C_t_ values. Analytical sensitivities for parasite DNA were based on the Limit of Detection (LoD): the lowest DNA concentration detected consistently in three independent replicates. Clinical sensitivities for filter paper blots were based on the fraction of consistently positive results obtained for each sample using two to three independent replicates compared by *χ*^2^.

To compare C_t_ values for filter paper blots to those for parasite DNA standards, the units for the blood samples used to prepare the filter paper blots (asexual parasites per µl) were converted to pg DNA per qPCR based on 38.9 parasite genomes per pg parasite DNA, a 3 mm punch with 50 µl blood in each 10 mm diameter filter paper blot and the 6 µl of the 150 µl DNA eluate used for qPCR. Next, these results (pg DNA per qPCR) were used to solve for the y-intercepts (interpolated C_t_ values) of the regression lines for the *P. falciparum* 3D7 and Dd2 DNA standards. Finally, the interpolated C_t_ values were compared to the mean C_t_ values for filter paper blots using unpaired t-tests. Similar conversions were used to compare the sensitivity of qPCR for parasite DNA standards to its sensitivity for the DNA in smear-positive filter paper blots. Because blood was applied directly to filter paper blots and volumes (~ 50 µl) were estimated based on spot diameters (~ 1 cm), these comparisons aimed to understand how the qPCR results for parasite DNA standards relate to those for filter paper blots rather than provide accurate differences.

## Results

### In silico development and screening of candidate qPCR assays

Based on these studies, 13 candidate qPCR assays were selected for laboratory testing: nine developed previously and four developed during these studies (Tables 1 and 2). The template copy numbers for these assays ranged from 1 to 160, the AT content of DNA sequences amplified was < 80% and no cross-reactivity with host or pathogen DNA was detected using the NCBI Primer-Blast algorithm [11].

### Effects of different qPCR reagents and conditions on assay performance

Based on six tenfold dilutions of 3D7 *P. falciparum* DNA (from 10^+2^ to 10^−3^ pg DNA per qPCR), C_t_ values for qPCR assays were generally similar with reagents from three different manufacturers (Additional file 1). However, false-negative results for DNA standards were obtained using Quantabio reagents for the *crt* assay and confirmed by independent replicates in which neither fluorescent signals nor new amplicons were detected using qPCR and agarose gel electrophoresis. Because the qPCR assays performed well with NEB reagents, which were supplied as a master mix, subsequent studies were performed using the NEB reagent master mix.

Although most of the qPCR conditions examined with NEB reagents did not affect assay performance, increasing the annealing temperature (T_a_) from 50 to 60 ℃ did affect several TaqMan assays. At the higher T_a_ (60 ℃), C_t_ values increased for five assays (*crt*, *ldh*(b), *coxI*, *var*ATS and r364(b)) and one assay (*crt*76T) yielded false-negative results. In contrast, the C_t_ value for one assay (*cytb*) decreased. For the six remaining assays, there were no consistent differences in mean C_t_ values at 50 ℃ vs. 60 ℃. The optimal T_a_ for each assay was used to perform these studies.

As mentioned above, the *crt*76T assay yielded false-negative results using a T_a_ of 60° C, which may have been caused by the five mismatched nucleotides in the *crt*76T probe (TGAAT) vs. the Dd2 DNA template (ATTGA). Because the *crt*76T and *crt*76K assays were designed to hybridize with the polymorphic DNA sequences of *pfcrt*, those assays were excluded from further study. In contrast, the r364(a) assay yielded false-positive results with negative controls (nuclease-free water, human DNA). Based on gel electrophoresis, amplicons from those false-positive assays were smaller than those from true-positive assays (~ 100 vs. ~ 125 bp, Additional file 2). Because the false-positive results obtained with negative controls did not permit evaluation of the sensitivity of the r364(a) assay, the r364(a) assay was excluded from further study.

### Evaluation of qPCR assays based on C_t_ values for parasite DNA standards and filter paper blots from smear-positive asymptomatic individuals

**Fig. 1:**
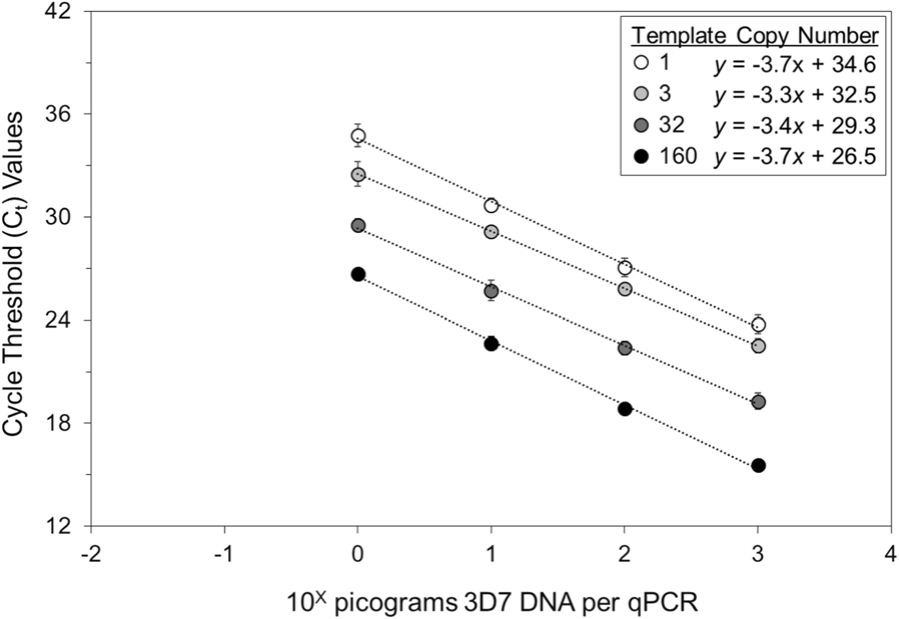
Effects of the amounts of 3D7 DNA and template copy number on C_t_ values. 3D7 *P. falciparum* DNA standards were tested by 2-way ANOVA for the effect of template copy number on the C_t_ value, which decreased as the amounts of parasite DNA and template copy numbers increased (mean C_t_ values and standard deviations are provided for 3D7 *P. falciparum* DNA from 10^°^ to 10^+3^ pg per qPCR, *p* < 0.001)

**Fig. 2:**
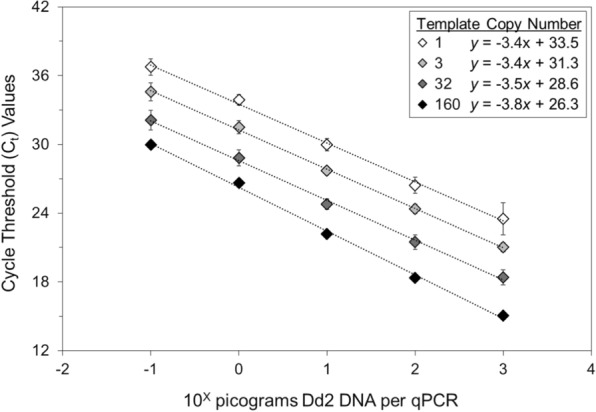
Effects of the amounts of Dd2 DNA and template copy number on C_t_ values. Dd2 *P. falciparum* DNA standards were tested by 2-way ANOVA for the effect of template copy number on the C_t_ value, which decreased as the amounts of parasite DNA and template copy numbers increased (mean C_t_ values and standard deviations are provided for Dd2 *P. falciparum* DNA from 10^−1^ to 10^+3^ pg per qPCR, *p* < 0.001)

**Fig. 3:**
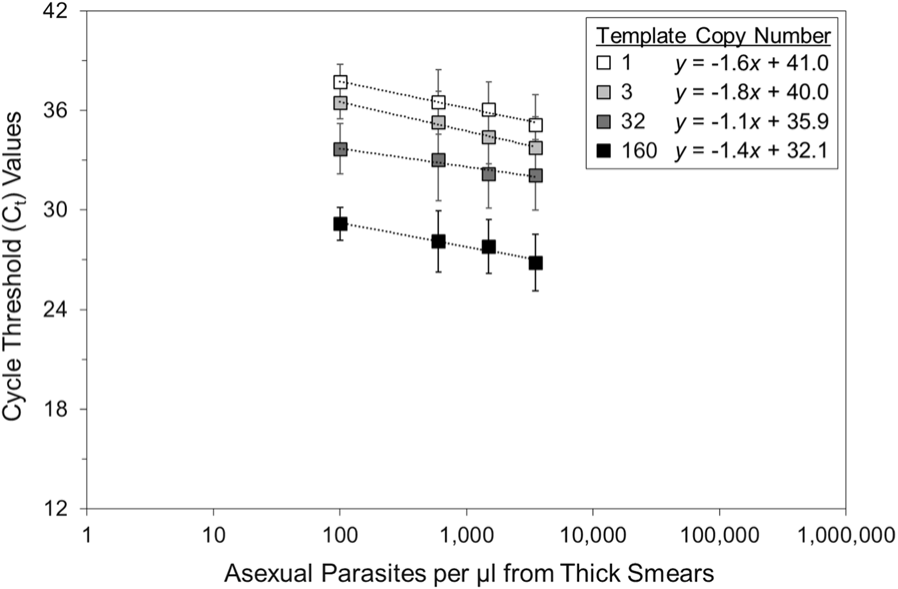
Effects of asexual parasite density and template copy number on C_t_ values. C_t_ values for smear-positive samples decreased as asexual parasite densities and template copy numbers increased (*p* < 0.001). Mean C_t_ values and standard deviations are grouped by their mean parasite densities and ranges of: 100 (≤ 200), 600 (201–999), 1500 (1000–1999) and 3500 (2000–5000) per μl. Note that the regression lines for the parasite DNA standards (Figs. 1, 2) were steeper than those for the filter paper blots (Fig. 3), consistent with DNA concentrations from 1000-fold (10^+3^) to 10,000- fold (10^+4^) for the DNA standards vs. 200-fold (10^+2.3^) for the filter paper blots

Of the two sets of 8 serially diluted parasite DNA standards and the 96 smear-positive filter paper blots examined, positive results were obtained for the qPCR assays with the four highest 3D7 DNA concentrations (10^+3^ to 10^0^ pg DNA per qPCR), the five highest Dd2 DNA concentrations (10^+3^ to 10^−1^ pg DNA per qPCR) and 51 of the 96 smear-positive filter paper blots (4 of 24 with ≤ 200 parasites per µl, 14 of 24 with 201–999 parasites per µl, 15 of 24 with 1000–1999 parasites per µl and 18 of 24 with 2000–5000 parasites per µl). Based on 2-way ANOVA testing, C_t_ values for the 3D7 and Dd2 parasite DNA standards (Figs. 1 and 2) and filter paper blots (Fig. 3) decreased as parasite DNA (i.e., amounts of DNA and parasite densities) and template copy numbers increased (*p* < 0.001).

Note that these results do not suggest confounding—because *p* values for the effects of parasite DNA concentrations and template copy numbers on the C_t_ values varied from 0.05 to 0.5 and the slopes of their regression lines were fundamentally similar (Additional file 3). In addition, the slopes of the regression lines for the parasite DNA standards (Figs. 1 and 2) were steeper than those for the filter paper blots (Fig. 3), consistent with DNA concentration ranges from 1000-fold (10^+3^) to 10,000- fold (10^+4^) for the DNA standards vs. 200-fold (10^+2.3^) for the filter paper blots.

### Evaluation of qPCR assays based on sensitivities for parasite DNA standards and filter paper blots of smear-positive asymptomatic subjects

**Fig. 4:**
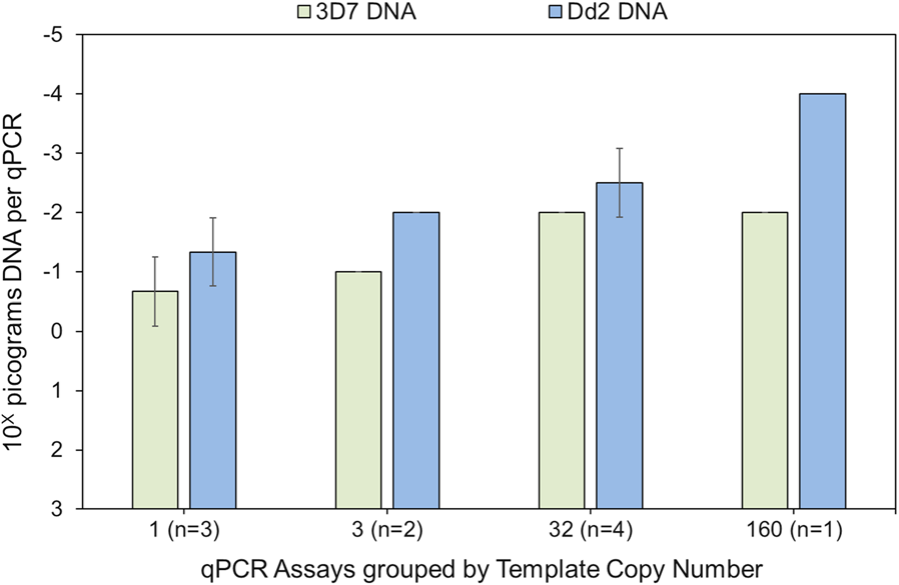
Limits of detection for 3D7 and Dd2 *P. falciparum* DNA by template copy number. The LoDs of qPCR assays for drug-susceptible (3D7) and –resistant (Dd2) *P. falciparum* DNA decreased (analytical sensitivities increased) as the template copy numbers of the qPCR assays increased (*p *< 0.001). Note that the TARE-2 assay (*n *= 160) yielded consistently positive results for the lowest concentration of Dd2 DNA tested initially (10^−4^ pg DNA per qPCR) but did not yield consistently positive results for an additional dilution of Dd2 DNA (10^−5^ pg DNA per qPCR)

Based on the drug-susceptible (3D7) and drug-resistant (Dd2) parasite DNA standards, the limits of detection (LoDs) for *P. falciparum* DNA decreased (sensitivities increased) as the template copy numbers increased (Fig. 4). Note that the TARE-2 assay yielded consistently positive results for the lowest concentration of Dd2 DNA tested initially (10^−4^ pg DNA per qPCR) but did not yield positive results for an additional dilution of Dd2 DNA (10^−5^ pg DNA per qPCR).

**Fig. 5:**
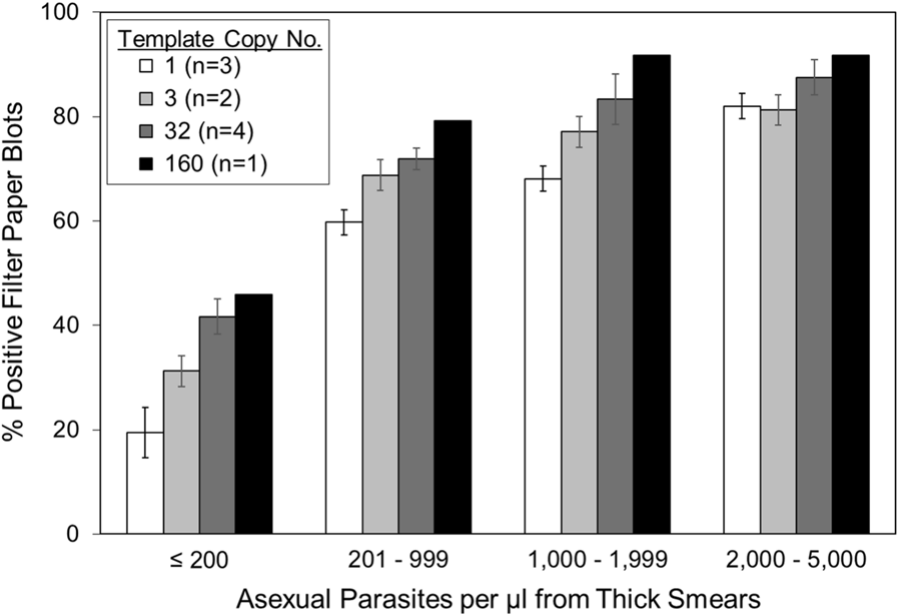
Detection of smear-positive samples by parasite count and template copy number. The clinical sensitivities of the qPCR assays for *P. falciparum* DNA reported here increased with parasite density and the template copy number in smear-positive, asymptomatic human subjects (*n *= 24 subjects per group, *p* < 0.001)

Based on filter paper blots stratified by parasite density, the sensitivities of qPCR assays for *P. falciparum* increased as parasite densities and template copy numbers increased (Fig. 5 and Additional file 4). Despite the gains in sensitivity due to increased template copy number, qPCR assays failed to detect more than half of the smear-positive filter paper blots at low parasite densities (≤ 200 asexual parasites per µl).

Based on *χ*^2^ testing, qPCR assays with template copy numbers from 32 (*n *= 4) to 160 (*n *= 1) were as expected more sensitive than assays with copy numbers of 1 (*n *= 3) (273/384 and 74/96 vs. 165/288, *p* < 0.001). Likewise, the qPCR assay with a template copy number of 160 (TARE-2) was more sensitive than the assays with template copy numbers of 3 (74/96 vs. 124/192, *p* < 0.05). In contrast, differences between the sensitivities of other qPCR assays (with template copy numbers of 1 vs. 3, 3 vs. 32 and 32 vs. 160) were not significant (*p* ≥ 0.1). These differences in significance were consistent with the differences between the template copy numbers of the assays: i.e., assays with greater differences between their template copy numbers (e.g., 1 vs. 160) were more likely to have differences between their outcomes than assays with smaller differences in template copy numbers (e.g., 1 vs. 3).

### Comparison of qPCR outcomes for DNA standards vs. filter paper blots

Based on conversions from parasites per μl to pg DNA per qPCR, the C_t_ values for filter paper blots were higher than those interpolated from the 3D7 and Dd2 DNA regression lines (mean C_t_ = 33.47 ± 3.17 vs. 28.73 ± 3.22 and 27.86 ± 3.09, respectively; *p* < 0.001). In addition, comparing the mean LoDs (analytical sensitivities) for 3D7 and Dd2 DNA standards using converted units (32 and 6 parasites per μl) to the clinical sensitivities for filter paper blots (fewer than half detected at ≤ 200 parasites per μl blood) suggest qPCR was more sensitive for the parasite DNA standards than the filter paper blots. Likewise, comparing the clinical sensitivities for filter paper blots using converted units (fewer than half detected at ≤ 0.9 pg DNA per qPCR) to the analytical sensitivities for 3D7 and Dd2 DNA standards (mean LoDs of 0.15 ± 0.30 and 0.03 ± 0.04 pg DNA per qPCR) suggests qPCR was more sensitive for the parasite DNA standards than the filter paper blots.

## Discussion

### In silico development and screening of qPCR candidate assays

The range of template copy numbers reported here for *P. falciparum*-specific qPCR assays is limited: from 1 to 160. In addition, because more assays were available for low template copy numbers (≤ 3 copies) than for higher template copy numbers, not all of the assays available for lower template copy numbers were examined in these studies and only one assay was available for ≥ 100 copies (TARE-2 for 160 copies). However, despite those limitations, the template copy numbers in the literature and those reported here permit an examination of the effects of template copy number on the sensitivity of qPCR for *P. falciparum* DNA based on DNA standards and smear-positive filter paper blots from asymptomatic subjects.

### Effects of different qPCR reagents and conditions on assay performance

Because pilot studies showed consistent qPCR assay performance with reagents from most manufacturers, the C_t_ values and sensitivities reported here are unlikely to have been confounded by the use of one set of reagents for qPCR testing (i.e., NEB reagents). In addition, because the annealing temperature was the most important factor for assay performance and was optimized for each assay, the results reported here are unlikely to have been confounded by the conditions used to perform the qPCR assays.

Based on the pilot studies, three assays were excluded from further examination. Two (*crt*76K and *crt*76T) were excluded because they were designed to hybridize with highly polymorphic DNA sequences (amino acids 72–76 in *pfcrt*) [13]. In addition, the r364(a) assay was excluded because they produced false-positive results with negative controls (water, human DNA). Those results were unexpected because false-positive results had not been reported previously with the r364(a) assay [17, 23, 24]. Although agarose gel electrophoresis (Additional file 2) suggested that false-positive results with the r364(a) assay could have resulted from hybridization of the probe to unintended smaller qPCR products, DNA contamination below the level of detection by agarose gel electrophoresis could not be excluded.

### Evaluation of qPCR assays based on C_t_ values for parasite DNA standards and filter paper blots from smear-positive asymptomatic subjects

Two-way ANOVA testing of C_t_ values for parasite DNA standards and smear-positive filter paper blots revealed that C_t_ values decreased as the amounts of parasite DNA and template copy numbers increased. In addition, because the slopes of the regression lines for the DNA standards were steeper than those for the filter paper blots (consistent with their greater range of DNA concentrations), those data are internally consistent.

### Evaluation of qPCR assays based on sensitivities for parasite DNA standards and filter paper blots from smear-positive asymptomatic subjects

The LoD results (Fig. 4) show that the sensitivities of qPCR for 3D7 (drug-susceptible) and Dd2 (-resistant) *P. falciparum* DNA increased with template copy number. However, there was an exception to this pattern for template copy numbers from 32 to 160. Within that range, the LoD decreased (sensitivity increased) for Dd2 DNA, but not for 3D7 DNA. Although this result raises the possibility of strain-specific differences in TARE-2 between 3D7 and Dd2 parasites, that hypothesis cannot be confirmed (or rejected) until comparable sequence data become available for the 3D7 and Dd2 parasite genomes.

The LoDs reported here for 3D7 DNA agree with those reported previously (*ldh*(a) [25], *18SrRNA*(a) [15, 26], *18SrRNA*(b) [16, 26], *var*ATS and TARE-2 [19]). However, because the LoDs for drug-resistant Dd2 DNA were lower than those for drug-susceptible 3D7 DNA, additional DNA was obtained to exclude technical variation in preparation of the DNA standards as a cause of those differences. The C_t_ values for the new Dd2 DNA standards were similar to those for the initial Dd2 DNA standards. Please note that the small number of replicates used to estimate the LoDs (*n *= 3) likely contributed to the differences observed [27]. In addition, because these results were obtained using DNA from only two parasite strains and the genetic diversity of *P. falciparum* varies across the globe, additional studies will be necessary to establish the generalizability (validity) of these observations.

The studies of smear-positive filter paper blots indicate that the clinical sensitivity of qPCR for *P. falciparum* in asymptomatic subjects increased with both parasite density and template copy number, although it was inadequate at lower parasite densities (≤ 200 per μl). However, it is difficult to compare these results to other studies of asymptomatic subjects because data for infected, asymptomatic individuals are rarely stratified by asexual parasite density. Because these studies were based on smear-positive asymptomatic children in Dangassa, additional studies will be necessary to determine whether they are generalizable to other malaria-endemic areas. Although the increased clinical sensitivity of qPCR assays with higher template copy numbers was most apparent with low-density infections (≤ 200 asexual parasites per µl), the most sensitive qPCR assays failed to identify more than half of the smear-positive filter paper blots at asexual parasite densities ≤ 200 per μl and may likewise fail to identify epidemiologically important reservoirs of infection in malaria-endemic areas.

### Comparison of qPCR outcomes for parasite DNA Standards and filter paper blots based on unit conversions

These results are consistent with other reports suggesting that parasite DNA from filter paper blots may have higher C_t_ values and lower PCR sensitivity than DNA extracted from whole blood—likely due in part to the smaller volumes of blood processed [28–30]. Furthermore, qPCR has been shown to have greater sensitivity for *P. falciparum* DNA in asymptomatic individuals than microscopy and RDTs based on the isolation of red blood cells (and *Plasmodium*) from larger volumes of whole blood (≥ 500 µl) to concentrate the DNA used for testing [31]. Because matching whole blood samples were not available for the filter paper blots examined in these studies, those potential limitations on the qPCR clinical sensitivities reported cannot be excluded.

### Other potential limitations on the clinical sensitivity of qPCR for filter paper blots from smear-positive asymptomatic subjects

As noted above, it is difficult to compare the clinical sensitivities reported in these studies to those in other studies because qPCR results for *P. falciparum* are rarely stratified by parasite densities based on microscopy. For that reason and because the clinical sensitivities reported here were based on the microscopy results and molecular methods used to examine filter paper blots, potential limitations related to those factors require further consideration: (1) false-positive blood smears, (2) DNA extraction method and (3) additional molecular approaches for detecting *P. falciparum* using filter paper blots.

1. *False*-*positive blood smears*: Because the limited clinical sensitivities reported could potentially have been explained by higher frequencies of false-positive smears based on the microscopy results for the Dangassa study site, the *18SrRNA*(a) assay [15] was used to cross-examine filter paper blots from smear-positive symptomatic children (*n *= 62) at a different study site (Dioro, Mali) where microscopy slides were read by a different team and infection was confirmed using rapid diagnostic tests for *P. falciparum* histidine-rich protein 2. Because the *18SrRNA*(a) assay results were similar for the filter paper blots from asymptomatic children at Dangassa and those from symptomatic children at Dioro with infections confirmed based on parasite antigen (25/48 vs. 17/24 with ≤ 999 parasites per µl and 38/48 vs. 34/38 with ≥ 1000 parasites per µl, p ≥ 0.1), the results of those studies suggest the clinical sensitivities reported are reproducible and unlikely limited to the microscopy results for the Dangassa study site.
  To test for false-positive smears caused by non-falciparum *Plasmodium*, filter paper blots negative for *P. falciparum* by qPCR were examined using a qPCR for *Plasmodium malariae* [32] (the most common non-falciparum plasmodial infection reported in this region of Mali [33, 34]). Because those results were negative, false-positive smears for *P. falciparum* from non-falciparum *Plasmodium* such as *P. malariae* are unlikely to explain the limited clinical sensitivities of the qPCR assays for *P. falciparum* reported here.
2. *DNA extraction method*: Because a number of methods have been used to extract DNA from filter paper blots, pilot studies were performed to examine the sensitivity of the ChargeSwitch^®^ method. The Chelex^®^-based method was selected for this comparison because it is thought to have greater sensitivity at low parasite densities [35–38]. However, based on smear-positive filter paper blots from symptomatic subjects (*n *= 66) in Dioro, Mali, the frequencies of positive PCR results were similar with Chelex^®^ and ChargeSwitch^®^: (1) 26/66 vs. 22/66, *p *= 0.5 for *18S* rRNA genes [15] and (2) 24/66 vs. 25/66, *p *= 0.8 for the repetitive element 364 (r364) DNA sequences [10]. In addition, the potential for negative results based on failed ChargeSwitch^®^ extractions was excluded by PCR amplifying human *IFNy* DNA for those extractions. Although these results suggest the sensitivity of the ChargeSwitch^®^ is similar to that of other methods, alternative methods that process larger volumes of blood from filter paper blots may warrant further study [39].
3. *Additional molecular approaches for detecting* *P. falciparum* *using filter paper blots*: Because pre-amplification of parasite DNA may improve the sensitivity of qPCR for *P. falciparum* in asymptomatic individuals [40], 12 filter paper blots were examined with and without pre-amplification using four qPCR assays with varying template copy numbers: *ldh*(b), *18SrRNA*(a), *coxI* and r364(b). Compared to specimens negative in every assay examined, results for these 12 specimens were expected to improve with pre-amplification because they were positive in the assay with the highest template copy number (TARE-2, *n *= 160), although they were negative in qPCR assays based on single copy DNA templates (*crt, ldh*(a)*, ldh*(b)). Based on the C_t_ values for specimens positive without and with pre-amplification (*n *= 14 per group), pre-amplification increased the amount of DNA template (mean C_t_ values fell from 36.8 ± 1.2 to 5.2 ± 1.5, *p* < 0.001). However, the fraction of positive samples was similar without and with pre-amplification (16/48 vs. 18/48, *p *= 0.83). These results indicate that pre-amplification of filter paper blots from field studies can increase the amount of parasite DNA template, but may not improve the fraction of the qPCR assays (samples or subjects) positive for *P. falciparum*.
  Because the generalizability of these findings is limited by the strategies used to amplify parasite DNA from filter paper blots, future studies should include alternative strategies such as DNA sequences from novel search algorithms [41], RNA sequences with higher template copy numbers [42, 43] and PCR-based methods linked to immunoassays [44].

## Conclusions

Based on parasite DNA standards and smear-positive filter paper blots from subjects with asymptomatic *P. falciparum* infection, these studies indicate the sensitivity of qPCR assays increases with their template copy number. However, despite the increased sensitivity of qPCR assays with higher template copy numbers, qPCR assays with still greater sensitivity will be necessary to identify asymptomatic *P. falciparum*-infected individuals with low parasite densities (≤ 200 per µl) in order to interrupt transmission.

## Supplementary information

**Additional file 1**: C_t_ values for *P. falciparum* 3D7 DNA using 3 different sources of reagents. These tables indicate that C_t_ values were fundamentally similar for 9 qPCR assays with template copy numbers from 1 to 56 using reagents from 3 different manufacturers.

**Additional file 2**: Gel electrophoresis image of r364(a) PET-PCR products. This gel electrophoresis image shows r364(a) PET-PCR products amplified for false-positive qPCR assays are smaller (~100 bp) than those for true-positive qPCR assays (~125 bp).

**Additional file 3**: Mean C_t_ values and standard deviations for DNA standards based on qPCR assays in ascending order by template copy number. In these tables, the mean C_t_ values and standard deviations of 3 independent replicates for the 10 qPCR assays examined are provided for *P. falciparum* 3D7 (Table A) and Dd2 (Table B) DNA from 10^-5^ pg to 10^+2^ pg DNA per qPCR. These data describe the linear dynamic range, precision and regression line (and thus PCR efficiency) of each assay.

**Additional file 4**: Clinical sensitivity of qPCR Assays for *P. falciparum* in 96 filter paper blots from smear-positive, asymptomatic subjects by parasite density and template copy number. This table shows the individual clinical sensitivities of 10 qPCR assays for *P. falciparum* in filter paper blots from smear-positive, asymptomatic subjects increased with parasite densities from thick smears and template copy number of qPCR assay.

## Data Availability

Data generated and/or analysed during the current study not provided in the published or additional material are available on request from the corresponding authors.

## Abbreviations

6-FAM: 6-Carboxyfluorescein fluorescent dye
BLAST: Basic Local Alignment Search Tool
Bp: Base pair(s)
C_t_: Cycle threshold
HEX: Hexachloro-Fluorescein fluorescent dye
IABkFQ: Iowa Black Fluorescent Quencher
LNA: Locked Nucleic Acid (probe)
LoD: Limit of detection
MGB: Minor Groove Binder (probe)
MGBEc: MGB Eclipse^®^ Fluorescent Quencher
PET-PCR: Photo-induced Electron Transfer PCR
*Pfcrt*: *Plasmodium falciparum* chloroquine resistance transporter (gene)
qPCR: Quantitative (real-time) PCR
RDT: Rapid diagnostic test
T_a_: Annealing temperature

## Definitions

C_t_ cycle threshold: Number of cycles required for the PCR fluorescent signal to exceed the threshold level of fluorescence (to distinguish samples with true-positive results from samples with only background fluorescence)
Infection: Bloodstream parasitaemia with Plasmodium, such as *P. falciparum*, with or without signs or symptoms of disease (chills, fever, headache, myalgias)
Malaria: Bloodstream infection with malaria parasites such as *P. falciparum*, *P. vivax, P. ovale* or *P. malariae* with signs or symptoms of disease
Limit of detection: Lowest concentration of a substance which can be distinguished from the absence of that substance
Locked nucleic acid (Probe): Modified RNA nucleotide in which the ribose is modified with a bridge connecting the 2’ oxygen and 4’ carbon to “lock” the ribose in the 3’-endo (North) conformation
Minor groove-binder (Probe): Molecule that binds to the minor groove of DNA to form stable complexes which increase the sequence specificity of PCR
Photo-induced electron transfer PCR: Use of self-quenching fluorogenic primers for PCR-based diagnosis of plasmodial infection
Rapid diagnostic tests: Lateral flow immunochromatographic assays used to identify persons with plasmodial infection by detecting parasite antigens in their blood
