# Supplementary material for: Template copy number and the sensitivity of quantitative PCR for Plasmodium falciparum in asymptomatic individuals
Source: Malar J. 2020 Aug 18;19:295. doi: 10.1186/s12936-020-03365-8 (PMC7436962; doi:10.1186/s12936-020-03365-8)
Supplement: Supplementary file 1 — Additional file 1: Ct values for P. falciparum 3D7 DNA using 3 different sources of reagents. These tables indicate that Ct values were fundamentally similar for 9 qPCR assays with template copy numbers from 1 to 56 using reagents from 3 different manufacturers. [file 12936_2020_3365_MOESM1_ESM.pdf]

Additional File 1.

C_t values for *P. falciparum* 3D7 DNA using 3 different sources of reagents

Table A. Invitrogen Platinum™ *Taq* with Green PCR buffer

**qPCR assay template copy number**	**C_t values for 10^X pg of 3D7 DNA per qPCR**					
	-3	-2	-1	0	1	2
crt [*n*=1]	*negative*	*negative*	37.9	35.8	32.0	28.2
*crt*76K [*n*=1]	*negative*	*negative*	*negative*	38.0	36.3	27.1
ldh(a) [*n*=1]	*negative*	*negative*	37.9	34.9	31.7	27.8
18SrRNA(a) [*n*=3]	*negative*	*negative*	39.2	33.3	29.9	26.5
r364(a) [*n*=14]	*negative*	*negative*	33.5	30.0	26.4	22.6
cytb [*n*=22]	*negative*	*negative*	32.9	28.8	25.4	21.8
coxI [*n*=22]	*negative*	36.4	33.1	29.6	25.8	22.4
*var*ATS [*n*=29]	*negative*	37.2	33.9	30.3	26.9	23.4
r364(b) [*n*=56]	*negative*	*negative*	34.9	30.9	27.4	24.0

Table B. New England Biolabs Luna® Universal Probe qPCR

**qPCR assay template copy number**	**C_t values for 10^X pg of 3D7 DNA per qPCR**					
	-3	-2	-1	0	1	2
crt [*n*=1]	*negative*	*negative*	37.0	32.9	29.5	25.9
*crt*76K [*n*=1]	*negative*	*negative*	*negative*	36.1	32.1	28.7
ldh(a) [*n*=1]	*negative*	*negative*	37.2	35.1	31.1	26.3
18SrRNA(a) [*n*=3]	*negative*	38.5	35.6	33.1	29.5	25.4
r364(a) [*n*=14]	*negative*	36.4	33.7	30.1	26.5	22.7
cytb [*n*=22]	*negative*	36.5	32.8	28.9	25.5	22.4
coxI [*n*=22]	*negative*	33.9	31.4	27.8	23.9	20.5
*var*ATS [*n*=29]	*negative*	38.3	33.8	30.1	26.7	23.0
r364(b) [*n*=56]	*negative*	37.0	34.1	30.7	27.1	23.6

Table C. Quantabio PerfeCTa qPCR ToughMix™

**qPCR assay template copy number**	**C_t values for 10^X pg of 3D7 DNA per qPCR**					
	-3	-2	-1	0	1	2
crt [*n*=1]	*negative*	*negative*	*negative*	*negative*	*negative*	*negative*
*crt*76K [*n*=1]	*negative*	38.2	35.6	34.4	30.8	27.8
ldh(a) [*n*=1]	*negative*	*negative*	38.8	36.0	32.5	29.4
18SrRNA(a) [*n*=3]	*negative*	*negative*	37.5	33.7	30.4	27.4
r364(a) [*n*=14]	*negative*	*negative*	37.7	33.4	29.8	25.7
cytb [*n*=22]	*negative*	38.4	34.5	30.4	27.1	23.6
coxI [*n*=22]	*negative*	38.8	34.4	30.3	26.9	24.4
*var*ATS [*n*=29]	*negative*	37.1	34.5	31.3	27.6	23.9
r364(b) [*n*=56]	*negative*	*negative*	36.8	32.9	29.5	26.3

These tables indicate that C_t values were fundamentally similar for 9 qPCR assays with template copy numbers from 1 to 56 using reagents from 3 different manufacturers. The *crt* assay, which yielded false-negative qPCR results with Quantabio reagents, was an exception to that generalization.
