# Supplementary material for: Template copy number and the sensitivity of quantitative PCR for Plasmodium falciparum in asymptomatic individuals
Source: Malar J. 2020 Aug 18;19:295. doi: 10.1186/s12936-020-03365-8 (PMC7436962; doi:10.1186/s12936-020-03365-8)
Supplement: Supplementary file 2 — Additional file 2: Gel electrophoresis image of r364(a) PET-PCR products. This gel electrophoresis image shows r364(a) PET-PCR products amplified for false-positive qPCR assays are smaller (~100 bp) than those for true-positive qPCR assays (~125 bp). [file 12936_2020_3365_MOESM2_ESM.pdf]

Additional File 2.

Gel electrophoresis image of r364(a) PET-PCR products

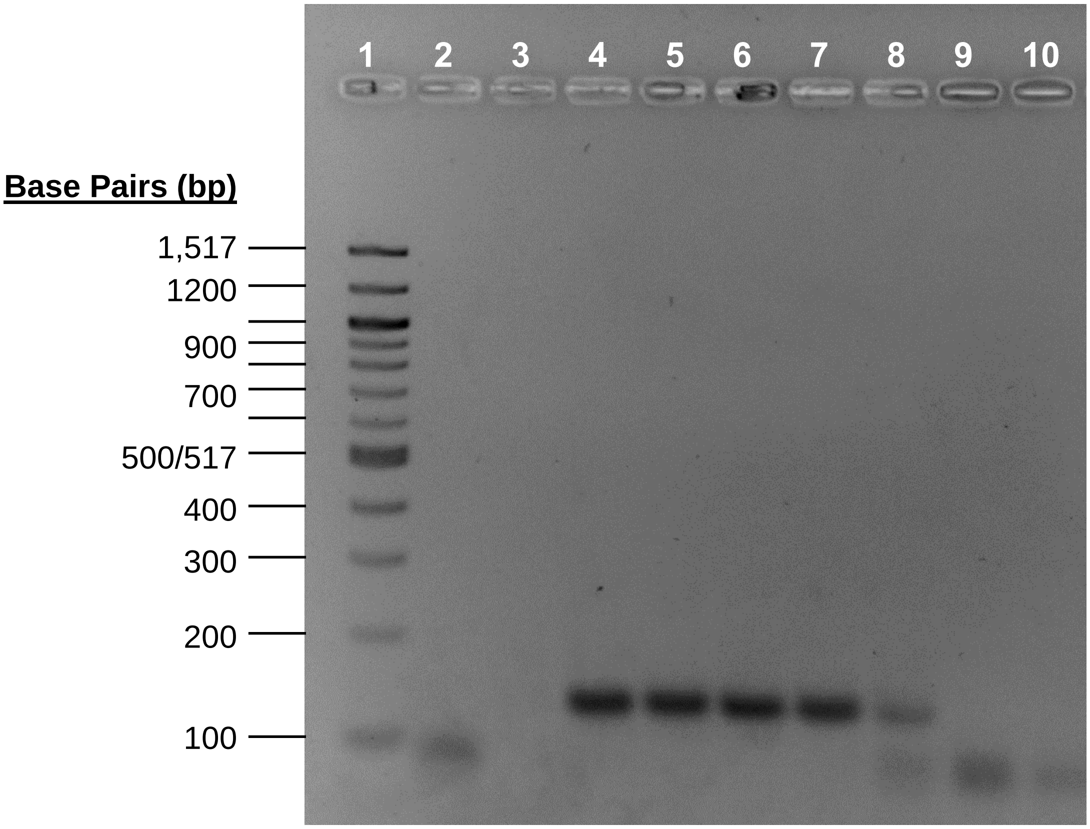

In this agarose gel, NEB Quick-Load Purple 100 bp ladder standards (#N0551) are in lane 1; the negative control for nuclease-free water in lane 2 showed a smaller amplicon (100 bp) and was therefore categorized as a false-positive qPCR assay (C_t-value=37); the negative control for human (non-plasmodial) DNA in lane 3 had no visible amplicons and was categorized as a true-negative qPCR assay (C_t-value=negative [>41]); the positive controls for parasite DNA in lanes 4 to 8 (10^{+2} to 10^{-2} pg DNA per qPCR) showed the larger amplicon (~125 bp) and were categorized as true-positive qPCR results (C_t-values=23, 27, 30, 34 and 37, respectively); the positive controls in lanes 9 and 10 (10^{-3} and 10^{-4} pg DNA per qPCR) showed only the smaller amplicon (~100 bp) and thus were categorized as false-positive qPCR assays (C_t=37 and 38).
