# Supplementary material for: Template copy number and the sensitivity of quantitative PCR for Plasmodium falciparum in asymptomatic individuals
Source: Malar J. 2020 Aug 18;19:295. doi: 10.1186/s12936-020-03365-8 (PMC7436962; doi:10.1186/s12936-020-03365-8)
Supplement: Supplementary file 3 — Additional file 3: Mean Ct values and standard deviations for DNA standards based on qPCR assays in ascending order by template copy number. In these tables, the mean Ct values and standard deviations of 3 independent replicates for the 10 qPCR assays examined are provided for P. falciparum 3D7 (Table A) and Dd2 (Table B) DNA from 10-5 pg to 10+2 pg DNA per qPCR. These data describe the linear dynamic range, precision and regression line (and thus PCR efficiency) of each assay. [file 12936_2020_3365_MOESM3_ESM.pdf]

Additional File 3.

Mean C_t values and standard deviations for DNA standards based on qPCR assays in ascending order by template copy number

Table A. *P. falciparum* 3D7 DNA standards

**qPCR assay**	**Mean C_t values ± standard deviations for 10^X pg of 3D7 DNA**							
	-4	-3	-2	-1	0	1	2	3
crt	*negative*	*negative*	*negative*	*negative*	34.7 ± 0.5	31.0 ± 0.1	27.7 ± 0.0	24.2 ± 0.1
ldh(a)	*negative*	*negative*	*negative*	37.4 ± 0.1	34.9 ± 1.2	30.7 ± 0.4	27.0 ± 0.0	24.0 ± 0.4
ldh(b)	*negative*	*negative*	*negative*	38.0 ± 0.8	34.7 ± 0.2	30.3 ± 0.1	26.4 ± 0.0	23.1 ± 0.0
18SrRNA(a)	*negative*	*negative*	*negative*	36.3 ± 0.5	32.7 ± 0.7	29.0 ± 0.3	25.7 ± 0.4	22.7 ± 0.6
18SrRNA(b)	*negative*	*negative*	*negative*	36.0 ± 0.7	32.4 ± 0.8	29.3 ± 0.2	25.9 ± 0.1	22.4 ± 0.1
cytb	*negative*	*negative*	37.8 ± 0.8	33.8 ± 0.5	29.9 ± 0.2	26.4 ± 0.2	22.6 ± 0.2	19.9 ± 0.6
coxI	*negative*	*negative*	36.8 ± 0.0	33.1 ± 0.2	29.4 ± 0.2	26.0 ± 0.0	22.6 ± 0.0	19.1 ± 0.0
*var*ATS	*negative*	*negative*	35.9 ± 1.0	33.8 ± 0.5	29.1 ± 0.2	25.6 ± 0.1	21.8 ± 0.0	18.8 ± 0.1
r364(b)	*negative*	*negative*	36.8 ± 2.1	33.5 ± 0.2	29.7 ± 0.2	24.9 ± 0.1	22.6 ± 0.1	19.3 ± 0.1
TARE-2	*negative*	*negative*	35.4 ± 2.0	31.1 ± 0.6	26.7 ± 0.1	22.7 ± 0.4	18.9 ± 0.1	15.6 ± 0.2

Table B. *P. falciparum* Dd2 DNA standards

**qPCR Assay**	**Mean C_t values ± standard deviations for 10^X pg of Dd2 DNA**							
	-4	-3	-2	-1	0	1	2	3
crt	*negative*	*negative*	negative	37.6 ± 0.5	34.1 ± 0.2	30.6 ± 0.1	27.2 ± 0.2	24.7 ± 1.6
ldh(a)	*negative*	*negative*	38.2 ± 0.7	36.5 ± 0.3	33.7 ± 0.8	30.0 ± 0.4	26.5 ± 0.3	23.5 ± 0.7
ldh(b)	*negative*	*negative*	*negative*	36.3 ± 0.5	33.9 ± 0.1	29.4 ± 0.1	25.6 ± 0.1	22.2 ± 0.1
18SrRNA(a)	*negative*	*negative*	37.9 ± 0.8	34.1 ± 0.3	31.2 ± 0.6	27.6 ± 0.2	24.2 ± 0.4	20.8 ± 0.2
18SrRNA(b)	*negative*	*negative*	37.7 ± 0.1	35.1 ± 0.8	31.8 ± 0.4	27.9 ± 0.4	24.5 ± 0.4	21.2 ± 0.5
cytb	*negative*	38.7 ± 0.5	35.9 ± 1.1	31.3 ± 0.3	28.5 ± 0.2	24.7 ± 0.2	20.9 ± 0.1	18.2 ± 0.4
coxI	*negative*	38.1 ± 1.8	34.6 ± 0.4	31.3 ± 0.3	28.0 ± 0.2	24.3 ± 0.1	20.9 ± 0.0	17.5 ± 0.1
*var*ATS	*negative*	*negative*	35.8 ± 0.7	32.5 ± 0.2	29.1 ± 0.1	25.5 ± 0.0	21.7 ± 0.0	18.7 ± 0.1
r364(b)	*negative*	*negative*	36.3 ± 0.7	33.3 ± 0.3	29.7 ± 0.2	24.6 ± 0.1	22.4 ± 0.2	19.2 ± 0.0
TARE-2	35.2 ± 2.4	36.2 ± 1.4	32.6 ± 0.6	30.0 ± 0.2	26.6 ± 0.1	22.2 ± 0.0	18.3 ± 0.1	15.1 ± 0.2

In these tables, the mean C_t values and standard deviations of 3 independent replicates for the 10 qPCR assays examined are provided for *P. falciparum* 3D7 (Table A) and Dd2 (Table B) DNA from 10^{-5} pg to 10^{+2} pg DNA per qPCR. These data describe the linear dynamic range, precision and regression line (and thus PCR efficiency) of each assay.
