# Supplementary material for: Template copy number and the sensitivity of quantitative PCR for Plasmodium falciparum in asymptomatic individuals
Source: Malar J. 2020 Aug 18;19:295. doi: 10.1186/s12936-020-03365-8 (PMC7436962; doi:10.1186/s12936-020-03365-8)
Supplement: Supplementary file 4 — Additional file 4: Clinical sensitivity of qPCR Assays for P. falciparum in 96 filter paper blots from smear-positive, asymptomatic subjects by parasite density and template copy number. This table shows the individual clinical sensitivities of 10 qPCR assays for P. falciparum in filter paper blots from smear-positive, asymptomatic subjects increased with parasite densities from thick smears and template copy number of qPCR assay. [file 12936_2020_3365_MOESM4_ESM.pdf]

Additional File 4.

Clinical sensitivity of qPCR Assays for *P. falciparum* in 96 filter paper blots from smear-positive, asymptomatic subjects by parasite density and template copy number

qPCR Assays	Template Copy No.	Asexual Parasites per µl by Microscopy (*n* = 24 subjects per group)				
		≤ 200	201–999	1,000–1,999	2,000–5,000	Totals
crt	1	4 (17%)	14 (58%)	17 (71%)	19 (79%)	54 (56%)
ldh(a)	1	4 (17%)	15 (63%)	16 (67%)	20 (83%)	55 (57%)
ldh(b)	1	6 (25%)	14 (58%)	16 (67%)	20 (83%)	56 (59%)
*18Sr*RNA(a)	3	8 (33%)	17 (71%)	19 (79%)	19 (79%)	63 (66%)
*18Sr*RNA(b)	3	7 (29%)	16 (67%)	18 (75%)	20 (83%)	61 (64%)
cytb	22	10 (42%)	17 (71%)	21 (88%)	22 (92%)	70 (73%)
coxI	22	11 (46%)	17 (71%)	19 (79%)	21 (88%)	68 (71%)
*var*ATS	29	10 (42%)	17 (71%)	21 (88%)	21 (88%)	69 (72%)
r364(b)	56	9 (38%)	18 (75%)	19 (79%)	20 (83%)	66 (69%)
TARE-2	160	11 (46%)	19 (79%)	22 (92%)	22 (92%)	74 (77%)

This table shows the individual clinical sensitivities of 10 qPCR assays for *P. falciparum* in filter paper blots from smear-positive, asymptomatic subjects increased with parasite densities from thick smears and template copy number of qPCR assay.
